# Validating OHIP-5 for Equitable Oral Healthcare in Nigeria

**DOI:** 10.1016/j.identj.2026.109668

**Published:** 2026-06-11

**Authors:** Abimbola M. Oladayo, Seyi John Akinloye, Mojisola Olujitan, Azeez Alade, Adeola T. Williams, Ajeigbe Olanrewaju, Oscar Rysavy, Shareef M. Dabdoub, Daniel J. Caplan, Taiwo A. Lawal, Azeez Butali, Folake B. Lawal

**Affiliations:** a Missouri School of Dentistry and Oral Health, A.T. Still University, Kirksville, Missouri, USA; b School of Dentistry, University of Alabama at Birmingham, Birmingham, Alabama, USA; c Department of Oral Pathology, Radiology and Medicine, College of Dentistry, University of Iowa, Iowa City, Iowa, USA; d Iowa Institute for Oral Health Research, University of Iowa, Iowa City, Iowa, USA; e Department of Child Oral Health, University of Ibadan and University College Hospital, Ibadan, Nigeria; f Department of Restorative Sciences, Minnesota Dental Research Center for Biomaterials and Biomechanics, School of Dentistry, University of Minnesota, Minneapolis, Minnesota, USA; g Division of Biostatistics and Computational Biology, College of Dentistry, University of Iowa, Iowa City, Iowa, USA; h Department of Preventive & Community Dentistry, University of Iowa, Iowa City, Iowa, USA; i Division of Pediatric Surgery, Department of Surgery, University of Ibadan and University College Hospital, Ibadan, Nigeria; j Department of Periodontology and Community Dentistry, University of Ibadan and University College Hospital, Ibadan, Nigeria; k Consortium for Advanced Research Training in Africa (CARTA), Nairobi, Kenya

**Keywords:** Oral health impact profile (OHIP-5), Healthcare access, Patient-reported outcome measures (PROMs), Low- and middle-income countries (LMICs), Yoruba language

## Abstract

**Introduction and aims:**

Healthcare access in low- and middle-income countries (LMICs) is constrained by structural and socio-cultural barriers, including language. Oral health, despite its importance to general health, is often overlooked in universal health coverage efforts. The Oral Health Impact Profile (OHIP-5) is a brief patient-reported outcome measure (PROM) for oral health–related quality of life (OHRQoL) but has not been adapted into indigenous Nigerian languages. This study aimed to translate and evaluate the Yoruba version (OHIP-5Yor), assess its psychometric performance using both English and Yoruba administrations, and explore its relevance for improving patient-centred oral healthcare access in LMICs.

**Methods:**

A cross-sectional survey of 143 adults was conducted at two dental centres in Ibadan, Nigeria. The OHIP-5 was translated using a forward–backward approach with pilot testing. Psychometric evaluation included internal consistency, inter-item correlation, convergent validity, and confirmatory factor analysis.

**Results:**

Of the 143 participants, 52 completed the Yoruba version and 91 the English version. The mean OHIP-5 score was 6.6 ± 4.3. The instrument showed acceptable internal consistency (Cronbach’s α = 0.67) and supportive construct validity. Structural validity indices, however, indicated that the factorial structure requires further evaluation in larger and more diverse samples.

**Conclusions:**

The findings provide preliminary support for the reliability and validity of the OHIP-5Yor as a culturally adapted tool for assessing OHRQoL among Nigerian adult dental patients. Beyond psychometric evaluation, the instrument may help address an important equity gap by enabling non-English speakers to participate more fully in oral health assessment, thereby supporting patient–clinician communication and highlighting unmet needs. Incorporation of such tools into routine care and public health surveillance represents a potential, scalable approach to strengthening oral healthcare access in low- and middle-income countries (LMICs).

**Clinical relevance:**

The OHIP-5Yor enables brief, culturally appropriate assessment of patient-perceived oral health impacts and may support more equitable oral healthcare delivery in multilingual settings.

## Introduction

Access to healthcare in low- and middle-income countries (LMICs) is constrained by economic, infrastructural, and socio-cultural barriers. Oral health is integral to overall health and well-being, yet remains underrepresented in universal health coverage (UHC) agendas, and patient perspectives are rarely captured systematically in routine care. In this context, culturally and linguistically adapted patient-reported outcome measures (PROMs) can strengthen patient–clinician communication, reveal unmet needs, and generate evidence to inform equitable service delivery and policy.^1^

The Oral Health Impact Profile (OHIP) is one of the most widely used measure of oral health–related quality of life (OHRQoL).^2^, ^3^, ^4^ Developed from Locker’s model, OHIP has been shortened from 49 items to 14 and, most recently, to a 5-item ultrashort form (OHIP-5) that preserves the majority of information while substantially reducing respondent burden.^5^, ^6^, ^7^ This abridged version consists of 5-items quantifying quality of life (QoL) via four major dimensions: orofacial appearance, oral function, orofacial pain, and psychosocial impact,^8^ and has been validated in different languages and settings.^7^^,^^9^, ^10^, ^11^, ^12^, ^13^ It captures about 90% of the information from the original version while keeping only 10% of the contents.^9^ Its brevity improves feasibility and acceptability in busy clinics and surveys, particularly in resource-limited environments.^3^

Despite growing global use, OHIP-5 has not been evaluated in many LMIC contexts, including Nigeria, where oral disease burden is high and services are unevenly distributed.^14^^,^^15^ Psychometric properties of OHRQoL instruments can vary by population and culture, highlighting the need for cross-cultural adaptation and validation.^9^^,^^16^ Moreover, in Africa and other LMICs, PROMs are seldom integrated into routine oral healthcare or surveillance systems, and validated tools in indigenous languages are scarce, limiting equitable participation of non-English-speaking populations and obscuring access gaps that matter for policy and practice.^17^^,^^18^ Accordingly, this study aimed to translate and validate the OHIP-5 in Yoruba (OHIP-5Yor) among adult dental patients in southwestern Nigeria and to position this ultrashort measure as a practical enabler of patient-centred healthcare access. By reducing linguistic and administrative barriers to data collection, OHIP-5Yor is intended to support more inclusive assessment of OHRQoL in clinical and public health settings, complement clinicians’ evaluations, and provide decision-relevant evidence for oral health planning.

Nigeria is a multiethnic country with substantial cultural and linguistic diversity.^19^ validating OHIP-5 in Yoruba, one of the three major languages, provides baseline evidence for culturally appropriate OHRQoL assessment and a pathway to scale PROMs nationally, thereby informing service delivery, resource allocation, and UHC-aligned oral health policy.

## Methods

### Study design

A cross-sectional study was conducted at the Dental Clinics of the University College Hospital (UCH), Ibadan, and the Community Dental Center at Idikan, Oyo State, Nigeria in 2022. UCH is the principal tertiary dental referral centre in southwestern Nigeria, serving patients with complex treatment needs, whereas the Idikan Community Dental Center provides primary dental care for a predominantly low-income urban population. These sites were intentionally paired to capture variation in socioeconomic status and clinical severity within the Yoruba-speaking Ibadan catchment area. A convenience sample of adult patients presenting for routine examination or treatment was recruited. Based on recommendations of 2 to 20 participants per item for factor analysis of short scales,^10^^,^^20^^,^^21^ the required minimum for a 5-item instrument was exceeded (n = 143). Eligible participants were aged ≥18 years, able to communicate in English and/or Yoruba, and attending the clinic for routine dental care. Participants selected their preferred language version of the questionnaire. Individuals younger than 18 years or unable to participate meaningfully were excluded. The bilingual eligibility criterion was intended to facilitate participation among Yoruba speakers while maintaining instrument comparability. However, individuals with limited literacy in both languages may have been underrepresented, which may affect generalizability to the most linguistically marginalized groups. Because recruitment occurred among treatment-seeking adults in dental facilities, the sample likely overrepresents individuals with active symptoms or greater oral health concerns. Consequently, prevalence estimates of oral health–related quality of life (OHRQoL) impacts should be interpreted as clinic-based rather than representative of the broader population.

Ethical approval was obtained from the Institutional Review Board of the College of Medicine, University of Ibadan (UI/EC/22/0292). Participation was voluntary and informed consent was obtained prior to data collection.

### Translation of the OHIP5 into Yoruba

Two independent bilingual translators (with dental terminology familiarity) translated the English version of the OHIP-5 into Yoruba language using the forward-backward approach.^22^ An independent translator performed the forward translation to Yoruba, a separate bilingual translator, blinded to the original, performed the back-translation. An expert review process involving dental public health specialists and Yoruba language experts compared the original, translated, and back-translated versions to ensure semantic, conceptual, and experiential equivalence. Discrepancies were resolved through consensus. Pilot testing was subsequently conducted with 20 participants from the target population who were not included in the final study sample. Participants were asked whether any items were unclear and whether the wording accurately reflected their oral health experiences. Feedback from this process informed minor wording adjustments to improve clarity in items related to tooth loss, orofacial appearance, and perceived changes in food flavour. This process aimed to ensure linguistic clarity, cultural relevance, and conceptual consistency with the original OHIP-5 instrument.

### Measures

*Primary instrument*: The OHIP-5 assesses on four dimensions of impact (orofacial appearance, oral function, orofacial pain, and psychosocial impact), with subjects answering questions based on how frequently they experienced this on a 5-point Likert scale (0 = never; 1 = hardly ever; 2 = occasionally; 3 = fairly often; and 4 = very often). These responses resulted in a summary score ranging from 0 to 20, where a higher score indicates a higher perception of the negative impact of oral health problems.^13^

*Additional variables for validity testing and access context*: In addition to the OHIP-5 items, the survey questions captured socio-demographic information, oral symptoms, the perceived need for dental treatment, self-rating of general and oral health, satisfaction with the dental condition, diagnosis of the oral condition of the patient (presence of caries or periodontal disease as assessed by the dentist) and the proposed treatment plan (prophylactic or non-prophylactic). These variables supported convergent validity (e.g., correlations with self-rated health) and known-groups validity (e.g., differences by tooth loss, caries, periodontal status, treatment type), while situating OHRQoL findings within patterns of unmet need and service use relevant to healthcare access. The full English and Yoruba questionnaires are provided as supplementary material.

### Data collection

Trained and calibrated dentists administered the questionnaires and performed oral examinations prior to treatment. After providing informed consent, participants completed the questionnaire while seated in clinic waiting areas or operatories using either the English or Yoruba version according to language preference. Caries experience was assessed using the Decayed, Missing, and Filled Teeth (DMFT) index. For known-groups validity testing, only the D (decayed/untreated) component was used because it reflects current disease burden most directly associated with OHRQoL. Missing (M) and filled (F) components were captured separately through tooth count and treatment history variables; the use of the D component alone is acknowledged as a limitation.

Periodontal status was assessed using the Community Periodontal Index of Treatment Needs (CPITN). Additional variables recorded included number of teeth present (<20 vs ≥20), location of missing teeth (anterior vs posterior), and planned treatment type. Treatments were categorized as prophylactic (oral health education and routine scaling/polishing) or non-prophylactic (e.g., complex periodontal therapy, extractions, root canal therapy).

### Data analysis

Data analysis was conducted using R version 4.5.1 with the significance level set at 0.05.^23^ Categorical variables were summarized using frequencies and percentages, while continuous variables were reported as means and standard deviations. For descriptive purposes, OHIP-5 scores were dichotomized as 0 vs >0 to represent absence or presence of impact. To retain statistical precision, all reliability and validity analyses were conducted using continuous OHIP-5 scores. Reliability was assessed using Cronbach’s alpha with 95% bootstrap confidence intervals, calculated using the ltm package (v1.2-0),^24^ and average inter-item correlation. Convergent validity was assessed using the Spearman’s rank correlations and associated bootstrap confidence intervals between the OHIP5-Yor and global questions of perceived health status (oral and general). All bootstrap confidence intervals were calculated using 10000 bootstrap samples.

As the OHIP5-Yor response distribution was right-skewed with a significant Shapiro-Wilk test result, known groups validity was performed using the non-parametric Mann-Whitney U test. Differences between subjects were evaluated based on the number of teeth present (< 20 lt; 20 vs ≥ 20),^10^^,^^25^ location of missing teeth (anterior or posterior),^10^^,^^25^ caries experience using the D component of the DMFT caries index and dichotomized as presence vs absence of tooth decay,^26^ periodontal disease assessed as healthy periodontium with a CPITN score of 0 vs periodontal disease with CPITN scores > 0^27^ and treatment type assessed as prophylactic – which requires oral health education and routine scaling and polishing of the teeth vs non-prophylactic which included other treatments such as complex periodontal treatment, extraction, root canal therapy, etc. Rank-biserial correlations and associated 95% bootstrap confidence intervals were reported using the rcompanion package (v2.5.2).^28^

Structural validity was evaluated using confirmatory factor analysis (CFA) implemented in the lavaan package (v0.6-21) with a diagonally weighted least squares estimator (DWLS) appropriate for ordinal data. Although the OHIP-5 includes items representing multiple conceptual dimensions of oral health–related quality of life, it is designed and commonly used as an ultrashort summary measure of overall OHRQoL; therefore, a one-factor model was specified to reflect its intended scoring and to facilitate comparability with prior validation studies. Measurement invariance across English and Yoruba versions was assessed using multi-group CFA by comparing configural, metric (equal loadings), and scalar (equal loadings and thresholds) models across language groups using likelihood ratio tests. Model fit was evaluated using the Comparative Fit Index (CFI ≥ 0.95), Root Mean Square Error of Approximation (RMSEA ≤ 0.06), and Standardized Root Mean Square Residual (SRMR ≤ 0.08).^29^

## Results

### Participant characteristics

A total of 143 adults participated in the study, of whom 72 (50.3%) were female. Of the participants, 52 completed the Yoruba version of the OHIP-5 and 91 completed the English version; no participant completed both versions. The largest age group was 25 to 34 years (23.8%), and 51.0% were married. Most participants (63.6%) reported some college education or were college graduates. Self-perceived general health was most commonly reported as very good (39.2%), while 42.7% reported good oral health. Despite these ratings, 41.5% reported dissatisfaction with their current oral health status. Only 7.7% had fewer than 20 remaining teeth, and 48% were visiting the dental clinic for the first time – Table 1.

**Table 1:** Socio-demographic, self-reported general and oral health characteristics of the study participants.

Socio-demographic characteristics N = 143*	n (%)
**Age**	
18-24	30 (21%)
24-34	34 (23.8%)
35-44	23 (16.1%)
45-54	19 (13.3%)
54-64	21 (14.7%)
≥65	16 (11.2%)
**Gender**	
Female	72 (50.3%)
Male	69 (48.3%)
Prefer not to say	2 (1.4%)
**Level of education**	
None	4 (2.8%)
Less than High School	13 (9.1%)
High School	32 (22.4%)
Some College or College Graduate	91 (63.6%)
Other	3 (2.1%)
**Occupation**	
Dependent	31 (21.7%)
Unskilled	37 (25.9%)
Skilled	75 (52.4%)
**Marital status**	
Single	62 (43.4%)
Married	73 (51.0%)
Divorced	1 (0.7%)
Widowed	7 (4.9%)
**Participant health characteristics**	
**Self-reported general health status**	
Excellent	29 (20.3%)
Very good	56 (39.2%)
Good	45 (31.5%)
Fair	13 (9.1%)
Poor	0
**Self-reported oral health status**	
Excellent	5 (3.5%)
Very good	16 (11.2%)
Good	61 (42.7%)
Fair	52 (36.4%)
Poor	9 (6.3%)
**Satisfaction with current oral health**	
**Status**†	8 (5.6%)
Very satisfied	36 (25.4%)
Satisfied	35 (24.6%)
Neither satisfied nor dissatisfied	59 (41.5%)
Dissatisfied	4 (2.8%)
Very dissatisfied	
**Number of teeth remaining**	
<20	11 (7.7%)
≥20	132 (92.3%)
**Location of missing teeth**	
Anterior	17 (11.8%)
Posterior	46 (32.2%)
Both	26 (18.1%)
None	54 (37.8%)
**Caries**‡	26 (19%)
**Periodontitis**‡	77 (53.8%)
**Presence of comorbidities**‡	31 (23.0%)
**Smoking**	
Never smoker	126 (88.1%)
Current smoker	8 (5.6%)
Previous smoker	9 (6.3%)
**History of denture use**	7 (4.9%)

⁎ n (%).

† One missing observation.

‡ Nine missing observations.

#### OHIP-5 responses

The mean OHIP-5 summary score was 6.6 ± 4.3. Thirteen participants (9.1%) reported no oral health impact (score = 0), whereas 130 (90.9%) reported at least some impact (score ≥ 0). The most frequently reported impacts were painful aching in the mouth (75%) and difficulty chewing foods due to dental problems (70%). Lower frequencies were reported for feeling uncomfortable about oral appearance (59%), difficulty performing usual activities (39%) and reduced flavour perception in food (30%). Item-level means and prevalence of impacts are presented in Table 2.

**Table 2:** Oral health impact profile of the study participants (OHIP 5 responses).

OHIP-5 items	OHIP score Mean ± SD	OHIP 5 score > 0 n (%)
Difficulty chewing	1.89 ± 1.53	100 (70%)
Painful aching in your mouth	2.10 ± 1.50	107 (75%)
Felt uncomfortable	1.30 ± 1.33	84 (59%)
Felt that there has been less flavor	0.54 ± 1.01	43 (30%)
Had difficulty doing your usual jobs	0.80 ± 1.17	56 (39%)
OHIP-5 summary score	6.62 ± 4.31	130 (91%)

*Reliability*. Internal consistency for the combined English/Yoruba OHIP-5 was Cronbach’s α = 0.673 (95% CI: 0.555-0.756). The alpha if item deleted indicated that removing the appearance item increased α to 0.732. The average inter-item correlation was 0.31, consistent with a brief scale assessing a single construct^10^^,^^30^ – Table 3, Table 4. The correlation matrix showed a range of coefficients between −0.001 (between appearance and chewing) and 0.775 (between chewing and painful aching). Internal consistency results stratified by language group are presented in the supplementary material.

**Table 3:** Internal consistency of the OHIP-5 based on pooled responses from English and Yoruba administrations.

Questions	Scale mean if item deleted	Cronbach's alpha if item deleted
Difficulty chewing	4.75	0.563
Painful aching in your mouth	4.54	0.558
Uncomfortable about the appearance	5.34	0.732
Less flavor in your food	6.11	0.62
Difficulty doing your usual jobs	5.85	0.598
Cronbach's alpha		0.673

**Table 4:** Correlation matrix.

	Chewing	Aching	Appearance	Flavor	Jobs
Chewing	1				
Aching	0.775	1			
Appearance	−0.001	0.026	1		
Flavor	0.228	0.253	0.393	1	
Jobs	0.384	0.362	0.252	0.379	1

#### Convergent validity

OHIP-5 scores correlated negatively with self-perceived general health (Spearman’s ρ = −0.289, *P* < .001, 95% CI: [−0.433, −0.133) and self-perceived oral health (ρ = −0.330, *P* < .001, 95% CI: [−0.471, −0.172]), indicating that worse OHRQoL was associated with poorer perceived health (Supplementary material).

#### Known-groups validity

OHIP-5 scores differed significantly across several clinical groups (Table 5). Higher OHIP-5 scores were observed among participants with *posterior vs anterior tooth loss (P = .025), presence of untreated dental caries (P = .038) and non-prophylactic treatment needs compared with prophylactic care (P < .001).* No statistically significant difference in OHIP-5 scores was observed between participants with and without *periodontal disease (CPITN = 0 vs >0) (P = .207)*. Differences by *number of teeth present (<20 vs ≥20)* were also not statistically significant *(P = .075)*. These findings indicate that the OHIP-5 was able to discriminate between groups with different oral health conditions and treatment needs.

**Table 5:** Known groups validity.

Oral condition	N	Median	IQR	Rank-Biserial ρ (CI)	*P*
Teeth present				−0.323	
< 20	11	9.0	5.50	(−0.672,	.075
≥ 20	132	6.5	6.00	0.073)	
Location of missing teeth				0.414	
Anterior	13	4.0	3.00	(0.117,	.025*
Posterior	42	8.0	6.00	0.690)	
Caries				0.262	
Absent	108	6.0	6.00	(0.033,	.038*
Present	26	6.5	4.00	0.478)	
Periodontal disease				0.128	
Absent	57	6.0	5.00	(−0.071,	.207
Present	77	8.0	7.00	0.322)	
Treatment based on diagnosis				−0.482	<.001*
				(−0.679,	
Non-prophylactic	101	8.0	6.00	−0.266)	
Prophylactic	29	2.0	7.00		

⁎ Statistically significant at p < 0.05.

#### Structural validity

Both metric and scalar invariance were determined to hold between the Yoruba and English OHIP-5 translations, with non-significant likelihood ratio test results (*P* = .204 and *P* = .730, respectively). The 5-item, one-factor, scalar-invariant model showed improvement over the baseline model but did not meet conventional fit thresholds (CFI = 0.929; RMSEA = 0.187; SRMR = 0.172). Excluding the appearance item (standardized loading < 0.40) was subsequently considered. Metric and scalar invariance held (*P* = .108 and *P* = .939), with certain indices demonstrating improvement (4-item model: CFI = 0.984, RMSEA = 0.113, SRMR = 0.094). Each scalar-invariant model had a total McDonald’s omega of 0.81, exceeding the acceptable threshold of 0.7; however, RMSEA remained above the recommended ≤0.06 threshold and SRMR remained marginally above the ≤0.08 threshold in both models, indicating that the factorial structure requires further evaluation. Full CFA outputs for the scalar-invariant models, including the χ² statistic, degrees of freedom, standardized factor loadings, and thresholds, are provided in the supplementary material. These findings suggest that while the instrument demonstrates acceptable internal consistency and construct associations, its factorial structure warrants further examination in larger and more diverse samples. The observed model misfit may be attributable to several factors, including the modest sample size for confirmatory factor analysis, the ordinal nature of the response data, potential multidimensionality of the OHIP-5 construct, and residual effects of cross-cultural adaptation on item interpretation

## Discussion

The findings provide preliminary support for the reliability and validity of the OHIP-5Yor for assessing oral health–related quality of life (OHRQoL) among adult dental patients in southwestern Nigeria. In LMIC contexts where universal health coverage (UHC) goals are challenged by resource constraints and socio-cultural barriers, brief, culturally adapted PROMs offer a scalable way to capture patient experience, prioritize care, and inform resource allocation.

We observed a high prevalence of OHRQoL impacts (90.9%) and a mean OHIP-5 score of 6.6±4.3, with the most frequent impacts in orofacial pain and chewing difficulty. Differences in prevalence across studies are expected given variations in sampling frames and settings. Our estimates are comparable to those reported in clinic-based samples and higher than those from general-population studies, reflecting the greater disease burden among treatment-seeking adults.^13^^,^^16^^,^^26^^,^^31^^,^^32^ The prominence of pain and functional limitation aligns with previous research showing that untreated oral conditions significantly restrict daily functioning and productivity in LMIC contexts.^3^^,^^16^^,^^33^ Together with the substantial dissatisfaction reported and the high proportion of first-time clinic users, these findings suggest a considerable level of unmet oral health need that PROMs can help identify efficiently within routine services.

The validity criteria used in this study were selected a priori based on previous OHIP-5 validation studies. Internal consistency (Cronbach’s α ≈ 0.67) and the average inter-item correlation (0.31) indicate acceptable reliability for an ultra-short, unidimensional scale within this Yoruba-speaking population. Similar reliability estimates have been reported in other language adaptations of the OHIP-5, supporting the cross-cultural robustness of the instrument.^9^^,^^11^, ^12^, ^13^^,^^34^

Convergent validity was supported by negative correlations between OHIP-5 scores and self-rated oral and general health. Known-groups validity showed the expected gradients: anterior tooth loss, caries presence,^35^ and assignment to non-prophylactic treatment. Previous research has shown that tooth loss,^36^^,^^37^ periodontal disease,^38^ and caries are associated with the impairment of OHRQoL. Additionally, the distribution and the location of tooth loss play an essential role in the impact on the QoL.^36^ Although 53.8% of the respondents had some form of periodontitis, there was no association with the OHIP scores. The absence of a significant difference in the periodontal status might be explained by the length of time it takes for a substantial impact to occur in an individual, i.e., tooth mobility impacting chewing.^35^

Structural validity from CFA indicated improved fit when the lower-loading appearance item was excluded; however, RMSEA values exceeded conventional thresholds in both the 5-item and 4-item models. These suboptimal fit indices are consistent with the modest sample size and the potential sensitivity of DWLS-based RMSEA to ordinal data constraints in small samples. We therefore interpret these results cautiously, as preliminary indicators that the factorial structure of OHIP-5Yor may benefit from further refinement through larger, multi-site studies and cognitive debriefing of the appearance item.^39^ This was similar to findings from the English, Arabic, Spanish and German versions of the OHIP- 5.^9^^,^^10^ We interpret these reductions as analytic refinements rather than prescriptive item deletions. Future cognitive interviewing can probe comprehension of lower-loading items to optimize performance without sacrificing content coverage.

The Cross-cultural instrument development benefits from layered validation approaches that combine psychometric testing with qualitative methods such as expert review and cognitive debriefing. Recent work validating oral health knowledge and belief instruments in other non-English-speaking populations similarly demonstrates the value of integrating quantitative and qualitative approaches during instrument adaptation.^40^ Such frameworks provide useful methodological guidance for future refinements of OHIP-5Yor.

To our knowledge, this study represents the first validation of a widely used oral health PROM in a major indigenous Nigerian language. The findings demonstrate that an ultra-short instrument can perform acceptably while minimizing respondent burden, an important consideration for busy clinics and resource-constrained health systems. By complementing traditional clinical indicators with patient-reported experiences, OHIP-5Yor provides additional insight into the lived impact of oral disease. The instrument also has potential applications within primary care, maternal and child health, and non-communicable disease (NCD) programs where oral health intersects with systemic health. Furthermore, the brevity of the tool makes it suitable for integration into digital or mobile health platforms, enabling community-level screening and follow-up. The Yoruba version may also serve as a template for future cross-cultural adaptations into other major Nigerian languages to support national oral health equity.

### Implications for practice and policy

Routine implementation of OHIP-5Yor in clinical settings could facilitate patient-centred intake assessments, identify individuals experiencing high levels of oral health impact, and complement DMFT- and CPITN-based clinical evaluations with outcomes that matter to patients. Aggregated PROM data could also inform local service planning and national oral health strategies aligned with UHC and Sustainable Development Goal (SDG-3) frameworks.^41^ Digitizing the instrument (e.g., tablet- or SMS-based administration) could extend its reach to underserved rural populations and reduce language barriers at the point of care. Emerging studies using multi-group PROM analyses in dental settings highlight the importance of rigorous equivalence testing when tools are used across diverse populations. Future multi-site validation studies of OHIP-5Yor should therefore incorporate such approaches. Although this study focused on psychometric validation rather than implementation research, a potential deployment workflow could involve brief administration during patient registration or triage, rapid scoring by reception or nursing staff, and flagging of patients with higher scores for additional clinical attention. At a system level, aggregated data could be incorporated into clinic monitoring or public health surveillance systems to inform service planning. These implementation pathways are presented as potential applications and were not empirically evaluated in the present study.

### Strengths and limitations

This study contributes new evidence on the cross-cultural validation of an oral health PROM in Nigeria. The standardized translation process, inclusion of clinical indicators, and systematic psychometric evaluation strengthen the credibility and reproducibility of the findings. However, several limitations should be acknowledged. The use of a clinic-based convenience sample from a single urban region limits generalizability to the broader Yoruba-speaking population. Because participants were treatment-seeking adults, the sample likely overrepresents individuals with active oral disease and higher symptom burden, which may partly explain the high prevalence of OHRQoL impacts observed. Additionally, test–retest reliability was not assessed, and future work should examine the temporal stability of OHIP-5Yor scores over an appropriate interval. The bilingual eligibility criterion and clinic-based recruitment may also have excluded individuals with lower literacy or limited healthcare access, potentially underrepresenting the most marginalized populations. Future research priorities include multi-site and multi-language validation, incorporation of comprehensive clinical indices, and implementation studies evaluating how PROMs can be integrated into routine oral health service delivery and digital health systems.

## Conclusion

The OHIP-5Yor represents a culturally adapted, psychometrically acceptable tool for assessing oral health–related quality of life among Yoruba-speaking populations. By reducing linguistic barriers and enabling efficient patient-centred assessment, the instrument provides clinicians, researchers, and policymakers with a practical method for understanding the lived impact of oral disease. Such adaptations contribute to efforts to strengthen oral healthcare access and advance equitable health systems aligned with universal health coverage goals in LMIC settings.

## Conflict of interest

The authors declare no financial, economic, or professional interests that may have influenced the design, execution, or presentation of the outcomes of this study.

## Appendix Supplementary materials

Image, application 1

## Footnotes

Supplementary material associated with this article can be found in the online version at doi:10.1016/j.identj.2026.109668.
