# Supplementary material for: Validating OHIP-5 for Equitable Oral Healthcare in Nigeria
Source: Int Dent J. 2026 Jun 11;76(4):109668. doi: 10.1016/j.identj.2026.109668 (PMC13276555; doi:10.1016/j.identj.2026.109668)
Supplement: Supplementary file 1 [file mmc1.docx]

**Internal Consistency of OHIP-5**

| Questions | Cronbach's Alpha if Item Deleted | |
| --- | --- | --- |
|  | English | Yoruba |
| Difficulty chewing | 0.594 | 0.513 |
| Painful aching in your mouth | 0.607 | 0.479 |
| Uncomfortable about the appearance | 0.713 | 0.758 |
| Less flavor in your food | 0.655 | 0.563 |
| Difficulty doing your usual jobs | 0.615 | 0.574 |
| Cronbach's Alpha (95% CI) | 0.691  (0.529, 0.794) | 0.646  (0.459,  0.764) |

**Convergent Validity of OHIP-5**

|  | English | | Yoruba | |
| --- | --- | --- | --- | --- |
|  | Spearman’s ρ | p-value | Spearman’s ρ | p-value |
| General Health | -0.243  (-0.418, -0.046) | 0.020 | -0.359  (-0.595, -0.080) | 0.009 |
| Oral Health | -0.272  (-0.465, -0.058) | 0.009 | -0.434  (-0.635, -0.184) | 0.001 |

**Structural Validity, Full OHIP-5**

**Model Fit**

| χ2 | df | p |
| --- | --- | --- |
| 96.381 | 28 | <0.001 |

**Factor Loadings**

| Item | Estimate | Std. Error | z-value | P(>\|z\|) | Standardized (English) | Standardized (Yoruba) |
| --- | --- | --- | --- | --- | --- | --- |
| Chewing | 1.000 | — | — | — | 0.948 | 0.899 |
| Aching | 0.881 | 0.083 | 10.607 | <0.001 | 0.836 | 0.970 |
| Appearance | 0.298 | 0.089 | 3.333 | 0.001 | 0.283 | 0.260 |
| Flavor | 0.558 | 0.090 | 6.197 | <0.001 | 0.529 | 0.707 |
| Jobs | 0.631 | 0.083 | 7.552 | <0.001 | 0.598 | 0.675 |

**Thresholds**

| Item | Standardized (English) | Standardized (Yoruba) | Std. Error | z-value | P(>\|z\|) |
| --- | --- | --- | --- | --- | --- |
| Chewing 1 | -0.615 | -0.444 | 0.141 | -4.372 | <0.001 |
| Chewing 2 | -0.318 | -0.230 | 0.132 | -2.406 | 0.016 |
| Chewing 3 | 0.326 | 0.236 | 0.131 | 2.497 | 0.013 |
| Chewing 4 | 0.853 | 0.615 | 0.144 | 5.926 | <0.001 |
| Aching 1 | -0.713 | -0.630 | 0.143 | -4.996 | <0.001 |
| Aching 2 | -0.481 | -0.425 | 0.132 | -3.640 | <0.001 |
| Aching 3 | 0.088 | 0.078 | 0.122 | 0.722 | 0.471 |
| Aching 4 | 0.718 | 0.635 | 0.136 | 5.270 | <0.001 |
| Appearance 1 | -0.257 | -0.180 | 0.121 | -2.119 | 0.034 |
| Appearance 2 | 0.209 | 0.146 | 0.120 | 1.738 | 0.082 |
| Appearance 3 | 0.896 | 0.626 | 0.143 | 6.255 | <0.001 |
| Appearance 4 | 1.589 | 1.110 | 0.199 | 7.983 | <0.001 |
| Flavor 1 | 0.513 | 0.521 | 0.118 | 4.367 | <0.001 |
| Flavor 2 | 1.118 | 1.136 | 0.152 | 7.369 | <0.001 |
| Flavor 3 | 1.346 | 1.368 | 0.176 | 7.655 | <0.001 |
| Flavor 4 | 1.863 | 1.893 | 0.257 | 7.264 | <0.001 |
| Jobs 1 | 0.278 | 0.239 | 0.119 | 2.342 | 0.019 |
| Jobs 2 | 0.669 | 0.575 | 0.129 | 5.197 | <0.001 |
| Jobs 3 | 1.159 | 0.996 | 0.158 | 7.327 | <0.001 |
| Jobs 4 | 2.008 | 1.726 | 0.270 | 7.449 | <0.001 |

**Structural Validity, OHIP-5 without Appearance**

**Model Fit**

| χ2 | df | p |
| --- | --- | --- |
| 34.100 | 18 | 0.012 |

**Factor Loadings**

| Item | Estimate | Std. Error | z-value | P(>\|z\|) | Standardized (English) | Standardized (Yoruba) |
| --- | --- | --- | --- | --- | --- | --- |
| Chewing | 1.000 | — | — | — | 0.981 | 0.899 |
| Aching | 0.841 | 0.093 | 8.999 | <0.001 | 0.825 | 0.980 |
| Flavor | 0.433 | 0.092 | 4.721 | <0.001 | 0.425 | 0.664 |
| Jobs | 0.559 | 0.088 | 6.379 | <0.001 | 0.549 | 0.690 |

**Thresholds**

| Item | Standardized (English) | Standardized (Yoruba) | Std. Error | z-value | P(>\|z\|) |
| --- | --- | --- | --- | --- | --- |
| Chewing 1 | -0.647 | -0.394 | 0.141 | -4.574 | <0.001 |
| Chewing 2 | -0.318 | -0.194 | 0.132 | -2.402 | 0.016 |
| Chewing 3 | 0.354 | 0.216 | 0.132 | 2.681 | 0.007 |
| Chewing 4 | 0.896 | 0.546 | 0.146 | 6.141 | <0.001 |
| Aching 1 | -0.734 | -0.579 | 0.141 | -5.205 | <0.001 |
| Aching 2 | -0.476 | -0.376 | 0.131 | -3.632 | <0.001 |
| Aching 3 | 0.105 | 0.083 | 0.123 | 0.850 | 0.395 |
| Aching 4 | 0.755 | 0.596 | 0.138 | 5.478 | <0.001 |
| Flavor 1 | 0.500 | 0.520 | 0.115 | 4.336 | <0.001 |
| Flavor 2 | 1.117 | 1.161 | 0.152 | 7.362 | <0.001 |
| Flavor 3 | 1.340 | 1.393 | 0.176 | 7.634 | <0.001 |
| Flavor 4 | 1.848 | 1.922 | 0.257 | 7.199 | <0.001 |
| Jobs 1 | 0.268 | 0.224 | 0.118 | 2.275 | 0.023 |
| Jobs 2 | 0.680 | 0.569 | 0.129 | 5.295 | <0.001 |
| Jobs 3 | 1.177 | 0.985 | 0.159 | 7.414 | <0.001 |
| Jobs 4 | 2.034 | 1.701 | 0.269 | 7.564 | <0.001 |
